# Bioinspired Approaches and Their Philosophical–Ethical Dimensions: A Narrative Review

**DOI:** 10.3390/biomimetics10090602

**Published:** 2025-09-09

**Authors:** Louisa Estadieu, Julius Fenn, Michael Gorki, Philipp Höfele, Oliver Müller

**Affiliations:** 1 Cluster of Excellence livMatS @ FIT—Freiburg Center for Interactive Materials and Bioinspired Technologies, University of Freiburg, 79110 Freiburg, Germany; julius.fenn@livmats.uni-freiburg.de (J.F.); michael.gorki@livmats.uni-freiburg.de (M.G.); oliver.mueller@philosophie.uni-freiburg.de (O.M.); 2 Faculty of Philosophy, University of Freiburg, 79106 Freiburg, Germany; philipp.hoefele@frias.uni-freiburg.de; 3 Institute for Psychology, University of Freiburg, 79085 Freiburg im Breisgau, Germany; 4 Young Academy for Sustainability Research (YAS), Freiburg Institute for Advanced Studies (FRIAS), University of Freiburg, 79104 Freiburg, Germany

**Keywords:** ethics of bioinspired approaches, biomimicry, sustainability, climate crisis, human–nature relation

## Abstract

The environmental crisis demands transformative solutions on both technological and societal levels. Bioinspired approaches, which draw from the principles of natural systems, have emerged as a promising interdisciplinary framework to address these challenges. These approaches not only drive technological innovation but also provoke critical philosophical and ethical discourse, particularly in the field of biomimicry. Philosophical and ethical questions include: How can we ethically justify drawing inspiration from nature without exploiting it? How might a shift toward a bioinspired perspective alter our relationship with nature? How could a reorientation toward nature influence ethical frameworks and guide human behavior toward the environment? This narrative review systematically examines key philosophical and ethical perspectives within biomimicry, while focusing on potentials as well as limitations of these approaches to the environmental crisis. In doing so, it explores key perspectives such as “biomimetic ethics”, the “ontology of nature”, “bioinclusivity”, and the “naturalistic fallacy”.

## 1. Introduction

The Intergovernmental Panel on Climate Change (IPCC) has issued a stark warning: to limit global warming to 1.5–2 °C, global greenhouse gas (GHG) emissions must be cut by 45% by 2030 [1]. Meanwhile, a recent study by Richardson, Rockström, and colleagues (2023) revealed that Earth has already transgressed six of the nine planetary boundaries, pushing humanity well beyond its safe operating space [2,3,4]. As UN Secretary-General António Guterres recently warned, urgent action is needed to ensure that the 2030 Agenda for Sustainable Development does not become a symbol of missed opportunities and unfulfilled potential on a global scale [5]. Addressing this crisis requires rapid and far-reaching technological, societal, and systemic changes across disciplines and societal structures [6,7,8]. Beyond developing CO_2_-neutral technologies [9], redefining human perception and interaction with the non-human natural world is therefore essential for overcoming the traditional, ownership-centered paradigm, particularly dominant in Western societies [10].

One potential approach to addressing these challenges is the development of so-called bioinspired technologies, which are rooted in a circular economic framework characterized by a reciprocal “take and give back” philosophy [11,12,13,14]. Bioinspired approaches—including bionics, biomimetics, nature-based solutions, or biomimicry (for an overview, see Table 1)—seek to mimic, copy, or derive functional principles from biological systems, structures, and processes to inform technological innovation [15]. However, the discourse surrounding bioinspired technologies extends beyond their technical development to address sustainable, philosophical, and ethical, as well as societal, questions regarding bioinspired solutions. In this context, learning from nature is often associated with the aspiration for greater sustainability, not only by providing technological solutions but also by offering societal responses to the environmental crisis [16,17,18,19,20,21,22]. However, the assumed sustainability benefits of bioinspired technologies require critical evaluation on a case-by-case basis, as the so-called bionic or biomimetic “promise” [20] is not inherently guaranteed and, in some cases, may be considered both a technological and ethical fallacy [19,23,24,25]. This is particularly important for biomimicry, which explicitly seeks to address the challenges of climate change and promote alternative pathways to greater sustainability [26,27,28,29,30,31].

In the philosophical–ethical discussions on bioinspired approaches, biomimicry is particularly relevant. Defined as an “approach to innovation that seeks sustainable solutions to human challenges” involving social, philosophical and ethical, environmental, and economic dimensions [35], biomimicry is often characterized as an interdisciplinary design methodology that integrates philosophical reflections on human–nature relationships and sustainability sciences [17]. Thereby, the philosophical and ethical debates surrounding biomimicry raise important questions regarding the relationship between humans, technology, and nature, while also interrogating the conceptual foundations that shape our understanding of nature [21,26,27,28,29,30,31]. Key questions include:

- What normative assumptions underlie the transfer of biological systems to technological innovations [22,28]?
- How might a shift in our orientation toward nature transform our relationship with nature [29,36]?
- What kind of “nature” are we referring to in this context [21]?
- Could this shift lead to a new way of perceiving and valuing nature [26]?
- How might this reorientation reshape our ethical frameworks and, consequently, our behavior toward the natural world [37]?

These questions have a long-standing tradition in philosophical discourse, with roots tracing back to ancient philosophy. In contemporary debates, Janine Benyus plays a particularly significant role. In her book *Biomimicry: Innovation Inspired by Nature*, Benyus describes biomimicry as revolution, marking a shift from the exploitation of natural resources to an era based on exploration and learning from nature [26,37]. She identifies three key principles that underpin this shift: (a) nature as a model to “solve human problems”, (b) nature as a measure to “judge the ’rightness’ of our innovations”, and (c) nature as a “mentor” as it can guide human actions toward ecological sustainability [26]. Thereby, biomimicry is linked to sustainable design practices, as it uses nature’s ecological standards to assess the “rightness” of human innovations: after 3.8 billion years of evolution, nature has learned “what works, what is appropriate, and what lasts” [26]. Benyus argues that this shift towards nature might lead to a new appreciation of nature, allowing bioinspired approaches to align with Earth’s ecosystems [26].

Building on Benyus’s work, many philosophers addresses the ethical, societal, and normative dimensions of biomimicry [21,27,30,36,38,39]. Dicks formulates an entire philosophy of biomimicry, proposing a new conceptualization of nature to serve as its foundational ontology [21]. Mathews, in *Towards a Deeper Philosophy of Biomimicry*, discusses from an environmental ethical perspective how biomimicry transcends traditional dualisms and fundamentally transforms humanity’s relationship with nature [29,36]. She argues that biomimicry “moves us closer to the goal of planetary ecological integrity than the traditional environmental movement ever managed to do” [29]. Or as Dicks puts it: “The biomimicry movement is predominantly practical in orientation. Faced with what is now widely recognized as an ecological emergency, its main aims have been to promote biomimicry as a powerful response to that emergency, to develop tools and methodologies that facilitate biomimicry’s application to concrete problems in engineering and design, and—last but not least—to develop concrete solutions to these problems” [21]. Fisch takes this even further, describing biomimicry as a “social movement” with explicit political implications [40]. According to Fisch, biomimicry not only reflects on the relationship between nature and humans, but seeks to reshape it entirely:

“Biomimicry […] is more active than reflective, as it wants to establish an actual alternative relation with nature and a commensurate social ethics. At the same time, biomimicry distills the central theme of sustainability, which is that there is a ‘nature’ out there that we can learn from, whose so-called intelligent design holds the secret to the survival and future well-being of the human race”

[40].

Similar formulations on the transformative potential of biomimicry can be found on the website of the Biomimicry Institute with sentiments such as that biomimicry “is an inclusive community taking action to solve the challenges of climate change and biodiversity loss, the sense of disconnection from nature, and the impact of the “take-make-waste approach on nature” [35]. Moreover, biomimicry is said to offer a chance to make things right, to “embrace a systems view of our world and begin living within planetary bounds”, and “in practice [biomimicry] is dedicated to reconnecting people with nature, and aligning human systems with biological systems” [41]. In sum, proponents of biomimicry argue that it offers an innovative, eco-friendly perspective on engaging with nature, moving beyond the traditional mindset of domination and exploitation, and potentially providing solutions to the climate crisis.

To get the rich and complex debates into view, this paper presents a narrative review of key perspectives in the biomimicry debate, examining how discussions on biomimicry can contribute to addressing environmental challenges from a philosophical perspective. It particularly focuses on conceptual questions related to the notion of “nature” and explores how a reorientation toward nature could reshape ethical frameworks and human–nature relations. The paper is structured as follows: Section 3 analyzes key philosophical and ethical perspectives on the concept of nature and ethical questions regarding the human–nature relations within biomimicry, focusing on four key perspectives: (1) biomimetic ethics, (2) nature ontology, (3) bioinclusiveness, and (4) the naturalistic fallacy. Section 4 explores both the potential and the limitations of these perspectives in tackling environmental challenges and discusses further research directions.

## 2. Materials and Methods

For our narrative review, we conducted a literature search using various electronic databases, including Web of Science, Google Scholar, and JSTOR. We used the following combinations of search terms: (1) (“bioinspiration*” OR “bio-inspiration*” OR “biomimicry” OR “biomimetic*”) AND (“ethical*” OR “ethic*” OR “philosophy” OR “moral*”) AND (“concept* of nature”), (2) (“bioinspiration*” OR “bio-inspiration*” OR “biomimicry” OR “biomimetic*”) AND (“Anthropocene” OR “human-nature-relation*” OR “human-environment interaction*” OR “nature-culture interaction*”), (3) (“bioinspiration*” OR “bio-inspiration*” OR “biomimicry” OR “biomimetic*”) AND (“ontology” OR “ontological*”). Papers and books were included in the review if they were (1) written in English or German, (2) published in a peer-reviewed journal, published dissertation/thesis studies, or published reports, (3) addressed philosophical or ethical questions of biomimetics, bionics, bioinspiration, or biomimicry, (4) published over the past 31 years (1993–2024), and (5) specifically contributed to understanding how biomimicry can help address environmental challenges from a philosophical perspective.

## 3. Philosophical–Ethical Perspectives on the Concept of Nature and Human–Nature Relations Within Biomimicry

As biomimicry has grown in prominence, diverse philosophical and ethical perspectives have emerged discussing its potentiality and limitations. One perspective, biomimetic ethics, suggests that nature should serve not only as a resource but also as a model for ethical behavior [26,36,40,42]. This view advocates for drawing ethical principles from nature’s processes, emphasizing sustainability and interdependence. Another perspective, nature ontology, critiques the reductionist, technological interpretation of nature common in many biomimetic practices. Proponents of this line of argument call for a more holistic understanding of nature as a self-regulating, interconnected system [27,29,43,44]. Bioinclusiveness calls for biomimicry to move beyond human interests and engage with the protection of all species, advocating for a more symbiotic relationship with nature [31,37,38]. From a meta-perspective, critics warn against the naturalistic fallacy, which equates natural processes with ethical correctness, highlighting that not all natural systems are inherently sustainable or morally superior [24,28,37]. In the following section, we will discuss these perspectives in more detail.

The following provides an overview of these key perspectives on the philosophical and ethical implications of human–nature relations within biomimicry, with a particular focus on their environmental dimensions.

### 3.1. Biomimetic Ethics

**Figure 1:**
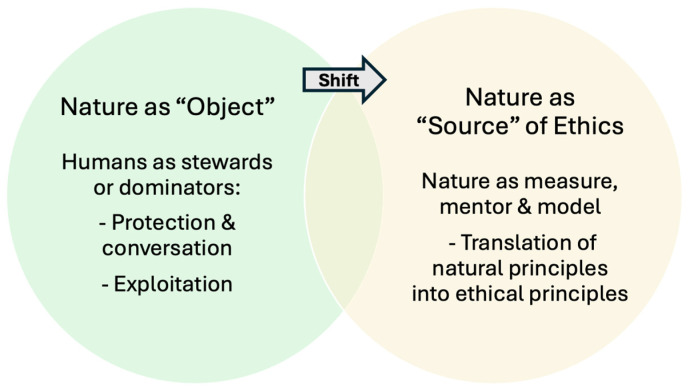
Conceptual visualization of the shift in perspective on nature within “biomimetic ethics”.

At its heart, “biomimetic ethics” contends that nature should not be regarded merely as an “object” to be either protected or exploited but rather as a vital source of ethical guidance and principles (see Figure 1) [26,40,42]. The term “biomimetic ethics” traces back to Henry Dicks, who critiques traditional environmental ethics for treating nature as an object rather than recognizing its potential as a model for ethical behavior [42]. Dicks argues that this anthropocentric view often neglects nature’s capacity to provide structures worthy of imitation. He proposes that, by observing natural systems, we can translate nature’s structures into a “set of generally applicable ethical laws and principles derived from Nature”, including biodiversity, reciprocity, and sustainability, which could guide more responsible and ecologically integrated human practices [42]. For example, the rich biodiversity of rainforests exemplifies diversity as a cornerstone of resilience in natural ecosystems. Dicks suggests that, just as biodiversity strengthens the resilience of ecosystems, cultural diversity could similarly enhance human societies’ adaptability, reinforcing the interconnectedness of human and ecological well-being. He states: “if it is true that all or almost all diversity in Nature is biodiversity, then it would need to be decided whether to include other forms of diversity (cultural, etc.) in the corresponding principle of human action” [42]. In a similar way, Mathews argues that philosophical bioinclusive ethics must transition from theoretical discussions to practical implementation [29,36]. Without this grounding, the philosophy of bioinspired approaches risks becoming merely a superficial design methodology rather than fostering a transformative cultural ethos.

“Biomimetic ethics” has faced some criticism, primarily for the risk of romanticizing nature and disregarding its more competitive, exploitative, and sometimes harmful dimensions [16]. Early in the debates, Rolston contended that the ethical principles proposed by biomimetic theories often rest on pre-existing moral assumptions [45]. He raises concerns about which natural laws should be emulated, as nature cannot be followed in an imitative ethical sense because nature itself might be amoral or even immoral [45]. Additionally, Blok suggests that searching for ethical guidance in nature risks oversimplifying the complexities of ecological dynamics, where conflict and competition are an integral aspect [27]. Another critique highlights that biomimetic ethics lacks a clear foundational framework and instead borrows from existing ethical systems [10].

### 3.2. Nature Ontology

“Nature ontology” critiques contemporary bioinspired practices for largely adopting a technological view of nature, reducing it to a purely mechanical or instrumental entity [37,43,46]. According to Zwart, biomimicry builds on a particular “view of nature (*Naturbild*), perceiving nature as an immense, outdoors laboratory” that can be emulated by tools and technologies developed in human-made laboratories [46]. Put differently, “nature is seen as an engineer involved in an enduring R&D [Research and Development] program to solve natural problems” [37], which limits a more holistic understanding of its complexities and interdependencies [38]. From this “technological” perspective, nature risks being reduced to mere functional principles, leading to a one-dimensional, reductionist view that fails to capture the ecological and intrinsic essence of natural processes beyond technological frameworks [17,24,43].

In contrast to this reductionistic view, proponents of “nature ontology” advocate for a broader philosophical and ecological understanding of nature, one that may prompt a shift in traditional epistemology, where nature is seen “as a source of ideas instead of goods” [26]. To clarify the “nature of nature”, they draw on philosophical insights from thinkers like Spinoza, Schelling, and Heidegger, as well as contemporary New Materialists such as Haraway, Latour, and Barad, to highlight key concepts of “living nature”, including causa sui (self-causation), autopoiesis (self-creation), and conatus (self-driven striving) or physis [21,29,43]. In doing so, they aim at formulating a new philosophical foundation for biomimicry, one that emphasizes an ecological, dynamic, and holistic understanding of nature rather than focusing solely on technological aspects [27]. This perspective encourages a more comprehensive understanding of natural entities, considering their entirety and interconnections with the environment—factors that should guide the development of bioinspired technologies. For example, Irwin and Baxter refer to Nancy Todd’s bioinspired living machine, stating, the “plant would be studied within the context of its environment in order to understand the dynamics of its ecosystem and provide clues about its fluid transformation into and out of being” [44]. From a philosophical standpoint, nature ontology underscores the intrinsic vitality of nature, redefines humanity as an embedded component of the natural world, and emphasizes the imperative for bioinspired design processes to integrate the ontological interdependence of technology and its environment.

Some authors, such as Tamborini, critique the oversimplified concept of nature within biomimicry and biomimetics [47]. Regarding Dicks’ concept of nature as *physis*, he argues that Dicks rightly highlights the importance of nature’s structures in biomimetics but his concept of natural form is overly focused on topology, neglecting the functional changes in living organisms. Instead, Tamborini proposes an epistemological foundation for bioinspired technology, focusing on the interaction and exchange of knowledge across disciplines involved in its design. This approach examines the practices connecting technical and biological fields, shifting from an ontological analysis to an exploration of the “grammar” of scientific practices. Drawing on Wittgenstein’s concept of philosophical grammar, Tamborini emphasizes the hidden rules that guide the use of concepts in these practices, highlighting the agency of both scientists and objects. This shift allows for a more nuanced understanding of bioinspired disciplines, revealing how the biomimetic principle is a multifaceted process with diverse, and sometimes contradictory, meanings [47]. Another point of critique might be that the proposed “new” ontological view is not entirely novel, as it resonates with ideas from philosophers like Spinoza, Kant, and Schelling, among others. As such, it carries significant metaphysical assumptions that require careful scrutiny, particularly in terms of the implicit normative and ontological presuppositions embedded within it [47]. Van der Hout et al. contend that this new ontological approach in biomimicry marks a shift from a “treasure-hunting” mentality to a “tutorial relationship” with nature [48]. However, they argue that both perspectives ultimately share a fundamental anthropocentric orientation, as they offer an implicit “reward” to humanity. According to them, this aspect merits further critical examination, particularly regarding the continuity of human-centered assumptions in both frameworks.

### 3.3. Bioinclusiveness

The perspective on bioinclusiveness, which is closely linked to biomimetics ethics and nature ontology, is rooted in the work of Freya Mathews [29,36]. In her view, biomimicry must be grounded in an ethical framework of bioinclusivity—“a principle that urges protection for all species”—since biomimicry alone does not inherently foster a more sustainable or ecological relationship with nature. Therefore biomimicry should expand the “moral” circle to include the interests of the members of the broader life system [36]. Key to bioinclusive ethics is the “ambiguity” of mimicry [29,36]: in the “human-centered” sense, mimicry involves replacing natural elements with artificial ones designed to follow natural principles but ultimately serving human needs. This form of biomimicry could result in a technicized “second nature” that displaces the original and reinforces human dominance. According to Mathews, the engineering of a second technological nature could further disconnect humans from natural systems, reduce incentives for biodiversity conservation, and undermine both ethical and ecological integrity [29]. In contrast, bioinclusiveness advocates for the genuine reintegration of humans into nature, recognizing all species as valuable and ensuring that they can collectively benefit from technological advancements. To ensure this, Mathews calls for an explicit ethical commitment to the protection of all species. Bioinclusive ethics comprises three interrelated principles: (1) a non-dualistic perception of humans and nature, (2) a non-technical understanding of nature, recognizing it not merely as a collection of material elements but as a realm of partly living entities with intrinsic values, (3) an ethical call for humanity to foster a synergistic relationship with nature, moving away from domination [29,36]. In this context, bioinclusivity is framed as an ethical principle advocating for the protection of all species. According to Mathews, without this ethical framework, bioinspired approaches risk becoming a tool of “ecomodernism” that ignores the ambivalence of uncritical acceleration of technological progress for sustainability.

Several scholars, including Veselova and Gaziulusoy, build upon Mathews’ concept of bioinclusive ethics or explore similar ideas [31]. Veselova and Gaziulusoy contend that design processes aimed at fostering “co-creative partnerships with nature” must move beyond the traditional “discourse of limits and constraints” in order to cultivate synergistic human–nature relationships and achieve positive environmental outcomes [31]. Similarly, Fisch [40] proposes a “relational” approach that nurtures a co-evolutionary relationship among humans, technology, and the environment, envisioning a future in which technology and nature co-exist as components of a shared, generative system rather than in opposition or subjugation.

While the concept of bioinclusivity is compelling, it encounters several challenges. First, its relationship to other environmental ethical frameworks, particularly biocentric approaches that already acknowledge nature’s inherent value, remains unclear. Second, the (metaphysical) assumptions regarding the inherent “value” of nature require explicit examination, as it is unclear what it truly means for nature to have value, a topic widely debated in environmental ethics [49,50]. Another critique that arises is the question of the scope of inclusion, which could lead to the prioritization of certain species. Additionally, broad interpretations of interconnectedness may encourage ethical relativism, raising critical concerns about responsibility and environmental justice.

### 3.4. Natural Fallacy

A significant critique of biomimicry is the potential to commit the naturalistic fallacy, which assumes that something is good simply because it is natural [24,28,37]. The naturalistic fallacy is a longstanding philosophical concept that, in Hume’s sense, refers to the error of deriving ethical, moral, or normative conclusions about what “ought” to be from purely factual statements about what “is” [51]. For example, a common critique in environmental ethics is that the concept of “value” is inherently anthropocentric, suggesting that nature lacks intrinsic value independent of human perception. Instead, value arises only when a subject evaluates something as, for example, “good”, “beautiful”, “cruel”, or even “sustainable”. However, whether nature is a “value-free” space is highly controversial [10,23,49,50,52,53]. Within biomimicry debates, some critics argue that using nature as an ethical or ecological benchmark risks committing the naturalistic fallacy by presupposing that what is natural is inherently good or superior [18,37]. This includes the belief that nature offers superior technical solutions, an ideal ethical framework, or inherent ethical values, all of which warrant critical scrutiny. Furthermore, critics argue that biomimicry’s focus on nature’s technical solutions can sometimes neglect the broader environmental, social, and ethical implications of applying such solutions in human contexts. While nature’s efficiency and adaptability are certainly valuable sources of inspiration, the direct transfer of these qualities to technology or design without careful consideration of the social and ecological impacts could risk reinforcing unsustainable practices, thereby perpetuating rather than resolving environmental issues.

## 4. Discussion

In our narrative review, we discussed four key philosophical and ethical perspectives within biomimicry regarding the concept of nature and human–nature relations (for an overview, see Table 2). We discussed “biomimetic ethics”, “nature ontology”, “bioinclusiveness”, and the “naturalistic fallacy”. These approaches examine the philosophical and ethical dimensions within biomimicry, focusing on how treating nature not only as a means to an end but as a “mentor”, “model”, or “measure” could offer a new concept of nature and thus new, less destructive ways of interacting with the natural world. These perspectives underscore the importance of challenging traditional dualisms, particularly the human–nature divide, and of exploring new frameworks that question one-sided power dynamics.

### Limitations and Future Research

While the narrative highlights four key philosophical and ethical perspectives within biomimicry regarding its environmental potential, it does not address other important perspectives, such as conceptual nuances, for example, different meanings of “mimicry”, or epistemic questions. For instance, scholars like Tamborini, Krohs, and Dicks have recently explored the varying meanings of “forms” and “functions” in biomimicry, as well as the socio-historical convergence of engineering and life sciences, to address the epistemology of biomimicry and biomimetics [30,43,47,55]. This line of research explores how these fields intersect and how biomimetics is influencing our understanding of knowledge and technology. Such research underscores the need for a more robust theoretical framework within biomimicry, as many studies tend to prioritize pragmatic, application-focused methods over addressing conceptual and epistemological questions [47,56]. Another point that has not been discussed is the shift from biomimicry to biohybridity, which represents a significant evolution in bioinspired approaches, reflecting a transition from nature serving as a model to a more integrated, co-creative relationship between natural and technological systems [57,58,59].

Furthermore, the field requires further investigation into potential consequences of excessive technological optimism, which may obscure critical systemic issues, such as social inequality and responsibility, that impact the envisioned transformative potential of biomimicry [46,59]. In this regard further research could discuss broader systemic challenges, such as the reliance on technological optimism that may obscure underlying issues like environmental degradation and different forms of inequality.

## 5. Conclusions

Our narrative review shows that biomimicry offers a promising interdisciplinary way of addressing the environmental challenges of our time. By drawing inspiration from nature on multiple levels, these approaches hold the potential to question traditional conceptions of nature and one-sided human–nature relations. Through a philosophical and ethical lens, we have explored several key perspectives that highlight both the potential and the challenges associated with this transformative potential as discussed in biomimicry.

## Figures and Tables

**Table 1:** Definitions of key terms in bioinspired approaches, including bioinspiration, biomimetics, biomimicry, bionics, nature-based solutions, and the biomimetic promise.

Term	Definition	Source
**Bioinspiration**	“Using phenomena in biology to stimulate research in non-biological science and technology”	[32]
**Biomimetics**	“Interdisciplinary cooperation of biology and technology or other fields of innovation with the goal of solving practical problems through the function analysis of biological systems, their abstraction into models, and the transfer into and application of these models to the solution”	[33]
**Biomimicry**	“Learning from the natural world by imitating or taking inspiration from nature’s designs and processes to solve human problems”; Nature as “model, measure and mentor”	[26,27,28,29,30,31,32,33,34]
**Bionics**	“Technical discipline that seeks to replicate, increase, or replace biological functions by their electronic and/or mechanical equivalents”	[33]
**Nature-based solutions**	“Nature-based solutions to societal challenges as solutions that are inspired or supported by nature, which are cost-effective, simultaneously provide environmental, social, and economic benefits, and help build resilience…”	[34]
**Biomimetic/Bionic promise**	“The biomimetic promise refers to higher resource efficiency, nature-inspired materials and processes, reduced risks and side effects, elegance, sophistication, and ecological fit, broadly speaking, to greater ecological sustainability” (transl. LE)	[19]

**Table 2:** Overview of philosophical and ethical perspectives within biomimicry.

Focus	Key Aspects	Critique	Source
Biomimetic Ethics	Nature as a source of ethical guidance Advocates for biodiversity, reciprocity, and sustainability Partnership with natural systems over stewardship	Risk of romanticizing nature and overlooking its competitive, exploitative, and sometimes harmful aspects Principles based on pre-existing moral assumptions	[16,26,27,29,40,42,45]
Nature Ontology	Critiques reductionist, technological perspectives of nature Advocates for a holistic and ecological understanding of nature	Oversimplification of natural systems Concerns regarding the metaphysical assumptions underlying the definition of nature	[29,37,38,43,46]
Bioinclusiveness	Protection for all species Non-dualistic perception of humans and nature Synergistic relationships and post-materialistic views Highlights risks of “second nature” displacing “first nature”	Unclear relationship to biocentric ethics Challenges include defining scope, operationalizing bioinclusivity in practical design Responsibility and possible ethical relativism	[29,31,36,40]
Natural Fallacy	Questions the assumption that “natural” equates to “good” Critiques the use of nature as a supreme ethical framework Highlights risks of oversimplifying ethical decisions	Inherent challenge in approaching nature without imposing human values	[18,24,37,39,54]
